# Mapping perceived training gaps in educational neuroscience among Brazilian teachers

**DOI:** 10.3389/fpsyg.2026.1923283

**Published:** 2026-08-21

**Authors:** Marcela Prado Siqueira, Analía Arévalo, Guilherme Lepski

**Affiliations:** 1 Department of Psychiatry, Medical School, University of São Paulo, São Paulo, Brazil; 2 Laboratory of Experimental Surgery (LIM26), Medical School, University of São Paulo, São Paulo, Brazil; 3 Department of Neurology, Medical School, University of São Paulo, São Paulo, Brazil

**Keywords:** Brazilian teachers, educational neuroscience, neuroscience literacy, self-perceived knowledge, teacher professional development, training needs assessment

## Abstract

Educational neuroscience has attracted substantial interest from teachers, but professional-development programs are often designed without a formal assessment of the difference between what educators believe they know and what they wish to learn. This secondary analysis of a cross-sectional survey examined perceived training needs in educational neuroscience among 204 active Brazilian teachers. Participants rated, on six-point scales (0–5), their self-perceived knowledge and their desire to learn more about 10 neuroscience-related topics. We defined a training-gap indicator as the within-participant difference between desired knowledge and self-perceived knowledge, ranging from −5 to +5. Positive values indicated a perceived need for further training. Overall self-perceived knowledge was 2.86 ± 1.04, whereas desire to learn was 3.88 ± 1.10, producing a mean global training gap of 1.01 ± 1.15. A positive global gap was present in 69.6% of participants. All 10 topics showed significant positive gaps after false-discovery-rate correction. The largest gaps concerned neurodevelopment and brain plasticity, the adolescent brain, dyslexia/dysgraphia/dyscalculia, learning disorders, and attention-deficit/hyperactivity disorder. Global training needs did not differ by school sector or teaching level and were not independently associated with age, teaching experience, gender, or disciplinary background. Training-format preferences differed significantly, with individual reading and remote modalities rated more highly than in-person formats, although the overall effect size was small. These findings suggest that perceived demand for educational- neuroscience training is broad rather than confined to specific teacher subgroups. A topic-prioritized, flexible, and scientifically grounded curriculum may therefore be more useful than narrowly targeted initiatives based only on demographic or institutional characteristics.

## 1 Introduction

Educational neuroscience seeks to translate evidence about learning, memory, neurodevelopment, emotion, and cognition into concepts that can inform educational practice. Teachers frequently seek such information to better understand students' behavior, learning difficulties, developmental variability, and classroom engagement. This

interest, however, develops in an environment in which scientific findings coexist with simplified commercial claims and persistent neuromyths. Misconceptions such as fixed learning styles, hemispheric dominance, or the belief that humans use only 10% of the brain remain common in educational settings and may divert time and resources toward ineffective practices (Dekker et al., 2012; Torrijos-Muelas et al., 2021; Simões et al., 2022).

Interest in neuroscience alone does not guarantee accurate knowledge. Teachers who actively read popular science material may still have difficulty distinguishing evidence-based explanations from persuasive but unsupported claims, particularly when neuro scientific terminology or brain images are used to lend credibility to weak educational arguments (Dekker et al., 2012; Torrijos-Muelas et al., 2021). Consequently, professional development should not merely increase exposure to neuroscience; it should improve critical appraisal, scientific literacy, and the ability to relate evidence to realistic pedagogical decisions.

Brazilian teachers work within highly heterogeneous institutional and professional contexts. Public and private school systems differ in infrastructure, workload, remuneration, and access to continuing education, while teachers at different educational levels follow distinct academic and professional pathways (Oliveira et al., 2016; Lemos and Fernandes, 2022; Langrafe Junior et al., 2025). Previous Brazilian research has also identified regional and educational-level differences in neuroscience knowledge and neuromyth endorsement (Simões et al., 2022). These inequalities are important, but subgroup comparisons alone do not indicate which content teachers themselves regard as most urgent.

Furthermore, Brazilian classrooms encompass marked socioeconomic, cultural, developmental, and institutional diversity, including students with different learning profiles and special educational needs. In daily practice, teachers are often expected to identify warning signs, adapt instruction, communicate with families, and initiate referrals, although diagnosis and clinical management remain the responsibility of specialized professionals. Initial teacher education in Brazil is heterogeneous, and neuroscience is not consistently included as a structured component of licensure curricula. When present, related content may appear indirectly within psychology, learning, development, or inclusive-education courses. This uneven preparation reinforces the value of assessing which neuroscience topics teachers themselves perceive as priorities for further training.

A practical training program requires a needs-assessment framework capable of integrating two dimensions: current self-perceived knowledge and desired future knowledge. A topic may deserve priority because knowledge is low, because interest is high, or because both conditions occur simultaneously. The difference between these ratings can be operationalized as a training gap. This approach provides an interpretable measure of perceived educational demand and permits comparison across topics and teacher characteristics.

The present study reports a secondary, question-driven analysis of a previously collected survey of Brazilian educators. Whereas the related primary report focused on variations in perceived neuroscience knowledge and interest across demographic, institutional, and professional groups, the present analysis centers on the within-person discrepancy between what teachers report knowing and what they would like to know. We aimed to: (1) quantify the global perceived training gap; (2) rank neuroscience-related topics according to training priority; (3) determine whether the gap varies by school sector, teaching level, age, experience, gender, or area of expertise; and (4) identify preferred formats for professional development.

## 2 Materials and methods

### 2.1 Study design and participants

This study is a secondary analysis of a cross-sectional, quantitative survey conducted among active Brazilian educators. A convenience sample of 204 teachers was recruited through professional educational networks and social media platforms. Eligibility was restricted to individuals who were actively teaching at the time of participation. Because some participants taught at more than one educational level, they were classified according to the highest level at which they taught.

### 2.2 Survey instrument and procedures

Data were collected through an anonymous online questionnaire hosted on Google Forms. The first section collected sociodemographic and professional information, including age, gender, years of teaching experience, school sector, highest teaching level, and area of expertise. Participants also indicated whether they considered neuroscience relevant to education and whether they were interested in further professional development.

The second section assessed 10 content domains: attention-deficit/hyperactivity disorder (ADHD), autism spectrum disorder (ASD), memory and learning, learning disorders, the child's brain, the adolescent brain, dyslexia/dysgraphia/dyscalculia, neurodevelopment and brain plasticity, socioemotional skills, and reading scientific articles/critical scientific literacy. For each topic, participants rated (a) how much they believed they currently knew and (b) how much they would like to know, using six-point scales from 0 to 5.

The final section assessed preferred training formats: asynchronous recorded classes, synchronous remote classes, in-person classes, individual reading, synchronous remote workshops, and in-person workshops. Each format was rated from 0 to 5.

The questionnaire was developed for this diagnostic study and was not subjected to a formal pre-study psychometric validation process. Its design was based on educator feedback obtained by the first author (an educator with more than 13 years of experience in both public and private Brazilian schools), as well as co-authors of our previous study (Simões et al., 2022), in which we surveyed more than 1,600 educators on neuroscience knowledge and neuromyth endorsement. Additionally, internal-consistency estimates were calculated *post hoc* for the three multi-item sections which yielded a Cronbach's alpha = 0.934 for the 10 self-perceived knowledge items, alpha = 0.950 for the 10 interest/relevance items, and alpha = 0.645 for the six training-format preference items. The first two sections therefore showed excellent internal consistency, whereas the format-preference section showed moderate consistency.

### 2.3 Training-gap indicator

For each participant and topic, a training-gap score was calculated as:

$$\begin{array}{l} \text{Training gap }=\text{ desired knowledge }-\text{ self-}perceived\;knowledge \end{array}$$

The score ranges from −5 to +5. Positive values indicate that the participant's desire to learn exceeds their self-perceived knowledge and were interpreted as a perceived need for training. A score of zero indicates equivalence between current and desired knowledge. Negative values indicate that self- perceived knowledge exceeds the desire for additional learning. For global analyses, we calculated each participant's mean self-perceived knowledge across the 10 topics, mean desired knowledge, and mean global training gap.

### 2.4 Ethical considerations

The study was conducted in accordance with the Declaration of Helsinki and was approved by the Research Ethics Committee of the Medical School of the University of São Paulo (CAAE 76331623.9.0000.0068). The first page of the online survey contained the informed-consent form, explained the aims of the study, and stated that no names or identifying information would be collected or stored. Only participants who provided electronic informed consent could access the questionnaire.

### 2.5 Statistical analysis

Because the original responses were recorded on ordinal six-point scales, inferential analyses prioritized non-parametric methods. Means and 95% confidence intervals were retained in tables and figures because they facilitate comparison and prioritization, while medians and interquartile ranges were also reported for global indicators.

Within each topic, self-perceived knowledge and desired knowledge were compared using the Wilcoxon signed-rank test. Ten topic-level *p*-values were adjusted using the Benjamini–Hochberg procedure. The proportion of participants with a positive gap and the rank-biserial correlation were reported as measures of practical relevance and effect size.

The global mean gap was compared across school sectors and teaching levels using Kruskal–Wallis tests, with epsilon-squared as the effect-size estimate. Pairwise Mann–Whitney tests with Holm correction were used when appropriate. Associations with age and teaching experience were assessed using Spearman correlations, whereas gender differences were examined using the Mann–Whitney test.

Areas of expertise were not mutually exclusive; therefore, each area was coded as a binary variable and initially examined with Mann–Whitney tests. The resulting *p*-values were adjusted using the Benjamini–Hochberg method. Separate univariable linear models with HC3 robust standard errors were additionally fitted to provide interpretable regression coefficients. A multivariable linear model with HC3 robust standard errors included age, years of experience, gender, and all areas of expertise simultaneously.

Training-format preferences were compared using the Friedman test because all participants rated all formats. Kendall's W quantified the overall effect size. *Post-hoc* paired comparisons used Wilcoxon signed-rank tests with Holm correction. All tests were two-sided, and *p* < 0.05 was considered statistically significant.

## 3 Results

### 3.1 Participant characteristics

The sample comprised 204 educators. Most participants were women (*n* = 164, 80.4%). Ninety-six worked in private schools, 90 in public schools, and 18 in both sectors. The largest teaching-level

groups were high school (*n* = 51), elementary school/early primary education (*n* = 47), and early childhood education (*n* = 39). See Table 1.

**Table 1:** Participant characteristics.

Characteristic	*N* (%)
Total	204
Women	164 (80)
Men	40 (20)
Private school	96 (47)
Public school	90 (44)
Both school sectors	18 (9)
Early childhood education	39 (19)
Elementary school	47 (23)
Middle school	25 (12)
High school	51 (25)
Higher education	36 (18)
Young-adult and adult education	6 (3)

### 3.2 Overall perceived training need

Across the 10 topics, mean self-perceived knowledge was 2.86 ± 1.04, whereas mean desired knowledge was 3.88 ± 1.10. The mean global training gap was 1.01 ± 1.15 points. Its median was 0.80 (interquartile range, 0.00–1.80), and 69.6% of participants had a positive global gap. Thus, for most teachers, desired knowledge exceeded perceived current knowledge. See Table 2 for all global indicators.

**Table 2:** Global indicators of self-perceived knowledge, desired knowledge, and training gap.

Indicator	Mean	SD	Median	IQR
Self-perceived knowledge	2.86	1.04	2.80	2.30–3.50
Desired knowledge	3.88	1.10	4.10	3.30–4.80
Global training gap	1.01	1.15	0.80	0.00–1.80

SD, standard deviation; IQR, interquartile range. Training gap = desired knowledge – self-perceived knowledge.

### 3.3 Topic prioritization

All 10 topics had positive mean training gaps, and every paired comparison remained significant after Benjamini–Hochberg correction (all adjusted *p* < 0.001). The largest gap concerned neurodevelopment and brain plasticity (1.41 points), followed by the adolescent brain (1.18), dyslexia/dysgraphia/dyscalculia (1.15), learning disorders (1.11), and ADHD (1.03). Reading scientific articles had the smallest mean gap (0.65), although desired knowledge remained higher than self- perceived knowledge. See Table 3 for all topic-specific estimates, proportions with a positive gap, adjusted *p*-values, and effect sizes.

**Table 3:** Topic-specific self-perceived knowledge, desired knowledge, and training gaps.

Topic	Knowledge mean ±SD	Desired mean ±SD	Mean gap	Positive gap %	Adjusted *p*	Effect size
Neurodevelopment and brain plasticity	2.55 ± 1.48	3.96 ± 1.37	1.41	63.2	< 0.001	0.94
Adolescent brain	2.47 ± 1.34	3.65 ± 1.48	1.18	54.4	< 0.001	0.88
Dyslexia, dysgraphia, and dyscalculia	2.51 ± 1.39	3.66 ± 1.36	1.15	58.3	< 0.001	0.87
Learning disorders	2.87 ± 1.27	3.98 ± 1.27	1.11	59.8	< 0.001	0.90
ADHD	2.87 ± 1.34	3.90 ± 1.28	1.03	54.9	< 0.001	0.89
Child's brain	2.78 ± 1.28	3.73 ± 1.43	0.95	52.9	< 0.001	0.77
ASD	2.85 ± 1.30	3.79 ± 1.32	0.94	54.9	< 0.001	0.79
Memory and learning	3.27 ± 1.14	4.16 ± 1.19	0.88	55.9	< 0.001	0.74
Socioemotional skills	3.24 ± 1.27	4.08 ± 1.14	0.84	51.5	< 0.001	0.79
Reading scientific articles	3.22 ± 1.35	3.87 ± 1.35	0.65	43.6	< 0.001	0.68

Adjusted *p*-values were obtained using the Benjamini–Hochberg procedure. Effect size is the rank-biserial correlation.

### 3.4 School sector and teaching level

**Figure 1:**
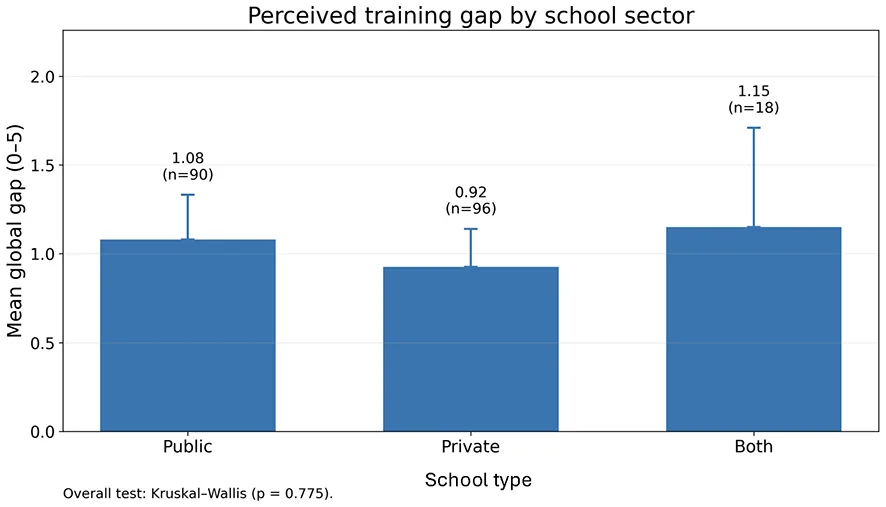
Global Global training gap according to school sector. Error bars represent 95% confidence intervals.

**Figure 2:**
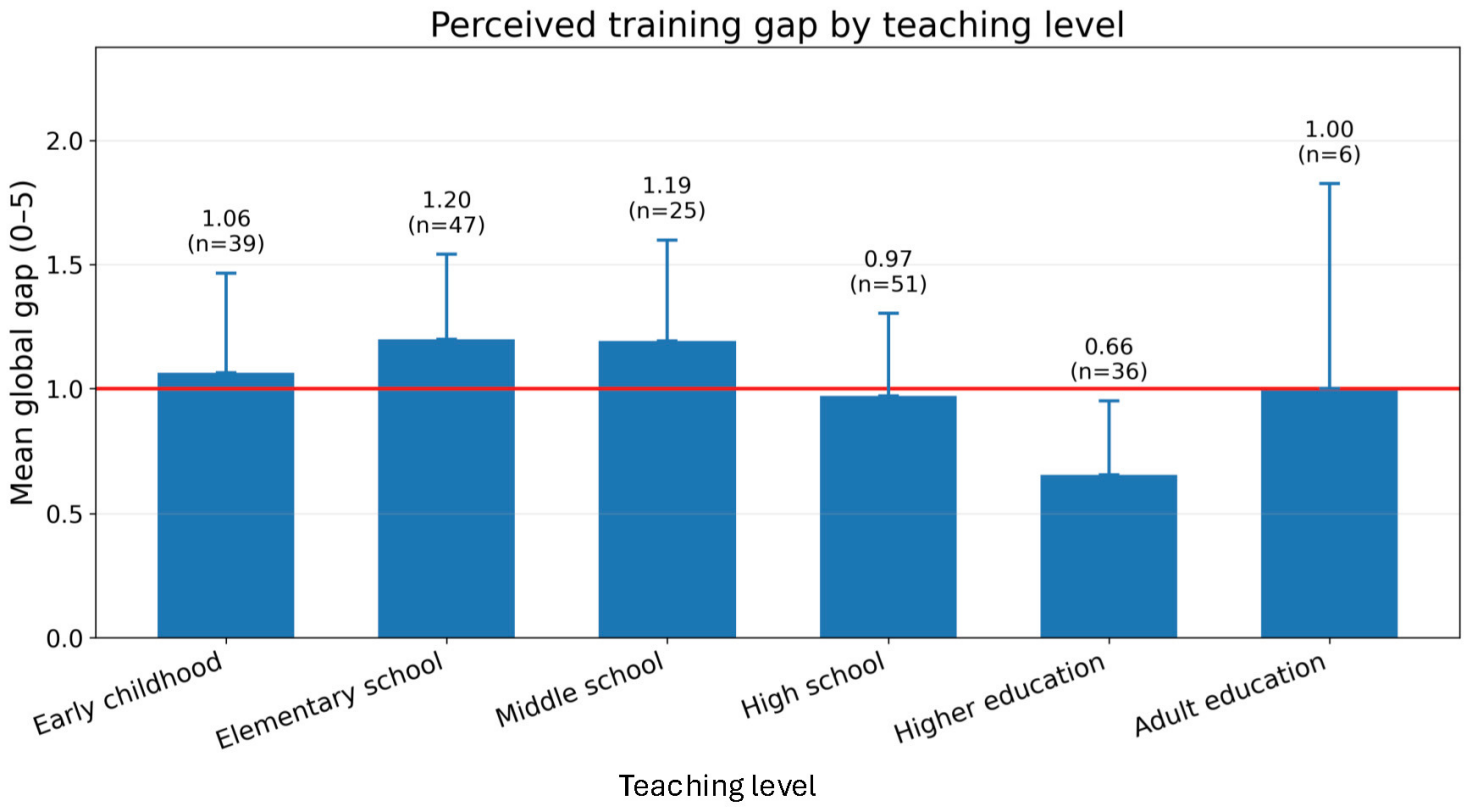
Global training gap according to the highest teaching level. Error bars represent 95% confidence intervals; the red horizontal line marks a mean gap of 1.0.

The global training gap did not differ across public, private, or dual-sector teachers (Kruskal–Wallis H = 0.47, *p* = 0.775; ε^2^ ≈ 0). It also did not differ significantly across the six teaching levels [H (5) = 4.40, *p* = 0.493; ε^2^ ≈ 0]. Although higher-education teachers had a numerically smaller gap, no pairwise comparison remained significant after Holm correction. Global training according to school sector and teaching level and shown in Figures 1, 2, respectively.

### 3.5 Age, experience, gender, and area of expertise

The global gap was not significantly associated with age (Spearman ρ = −0.084, *p* = 0.233) or years of teaching experience (ρ = −0.032, *p* = 0.654). Women and men had nearly identical mean gaps (1.01 and 1.02, respectively; Mann–Whitney *p* = 0.781). In unadjusted analyses, participants with a background in Linguistics, Letters, and Arts showed a larger gap than participants without that background; however, the corresponding non-parametric comparison did not remain significant after false-discovery-rate correction. No area of expertise was independently associated with the gap in the multivariable model.

**Figure 3:**
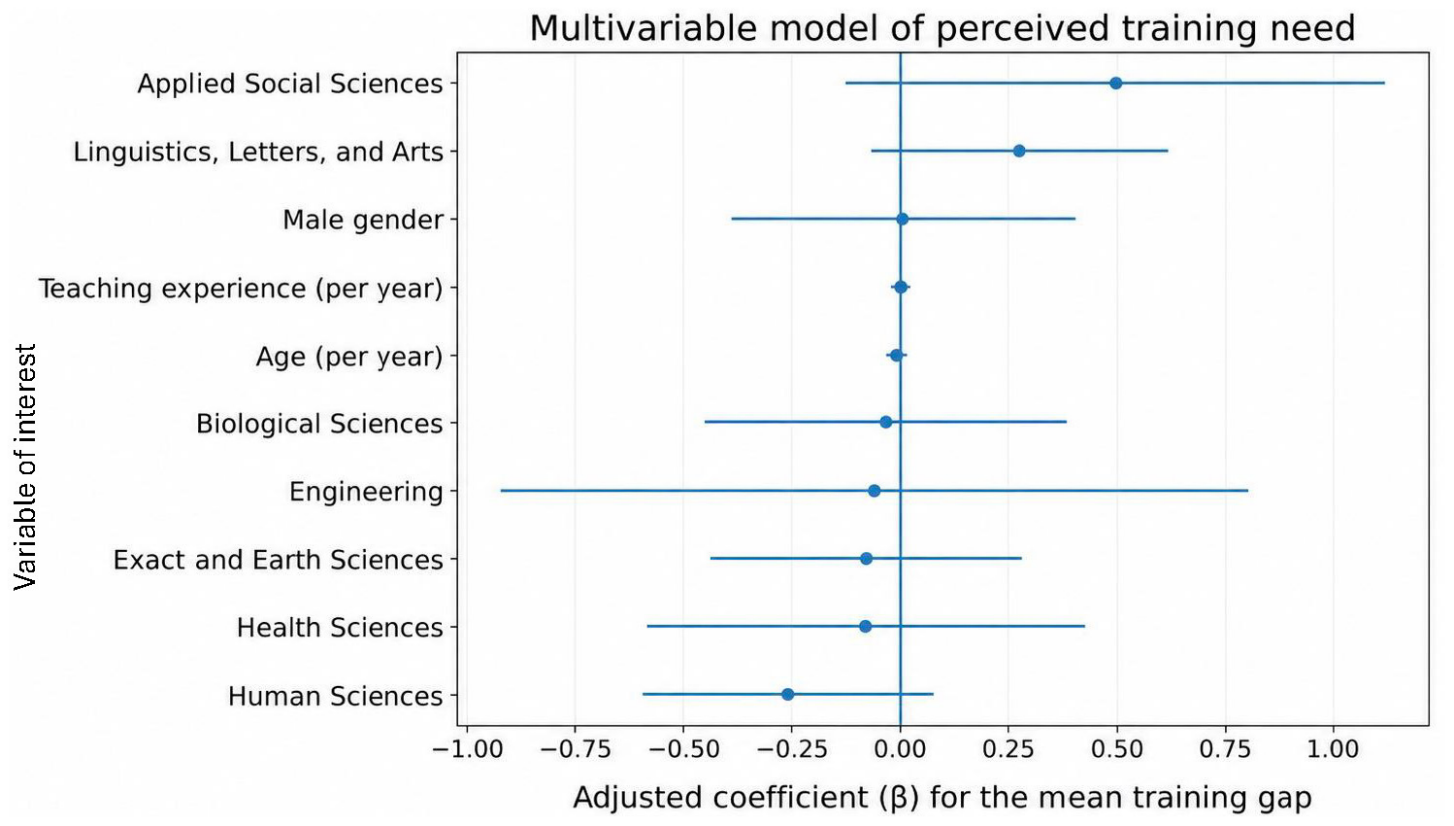
Adjusted coefficients from the multivariable model of the global training gap. Points are β coefficients and horizontal lines are 95% confidence intervals.

The multivariable model, including age, experience, gender, and seven disciplinary areas, explained 4.6% of the variance in the global gap (*R*^2^ = 0.046; adjusted *R*^2^ = −0.004) and was not globally significant (*F* = 0.94, *p* = 0.496). No adjusted 95% confidence interval excluded zero. Adjusted regression coefficients and confidence intervals are shown in Table 4 and Figure 3.

**Table 4:** Multivariable robust linear model of the global training gap.

Variable	Adjusted β	95% CI	*p*
Age, per year	−0.008	−0.031 to 0.015	0.492
Teaching experience, per year	0.001	−0.020 to 0.022	0.948
Male gender	0.006	−0.390 to 0.402	0.977
Linguistics, letters, and arts	0.274	−0.067 to 0.616	0.115
Exact and earth sciences	−0.078	−0.437 to 0.280	0.669
Human sciences	−0.260	−0.596 to 0.076	0.130
Biological sciences	−0.034	−0.452 to 0.384	0.874
Health sciences	−0.081	−0.585 to 0.424	0.754
Applied social sciences	0.496	−0.126 to 1.119	0.118
Engineering	−0.060	−0.922 to 0.803	0.892

HC3 robust standard errors; *n* = 201. Reference groups were female gender and absence of the respective disciplinary background.

### 3.6 Preferred training formats

**Figure 4:**
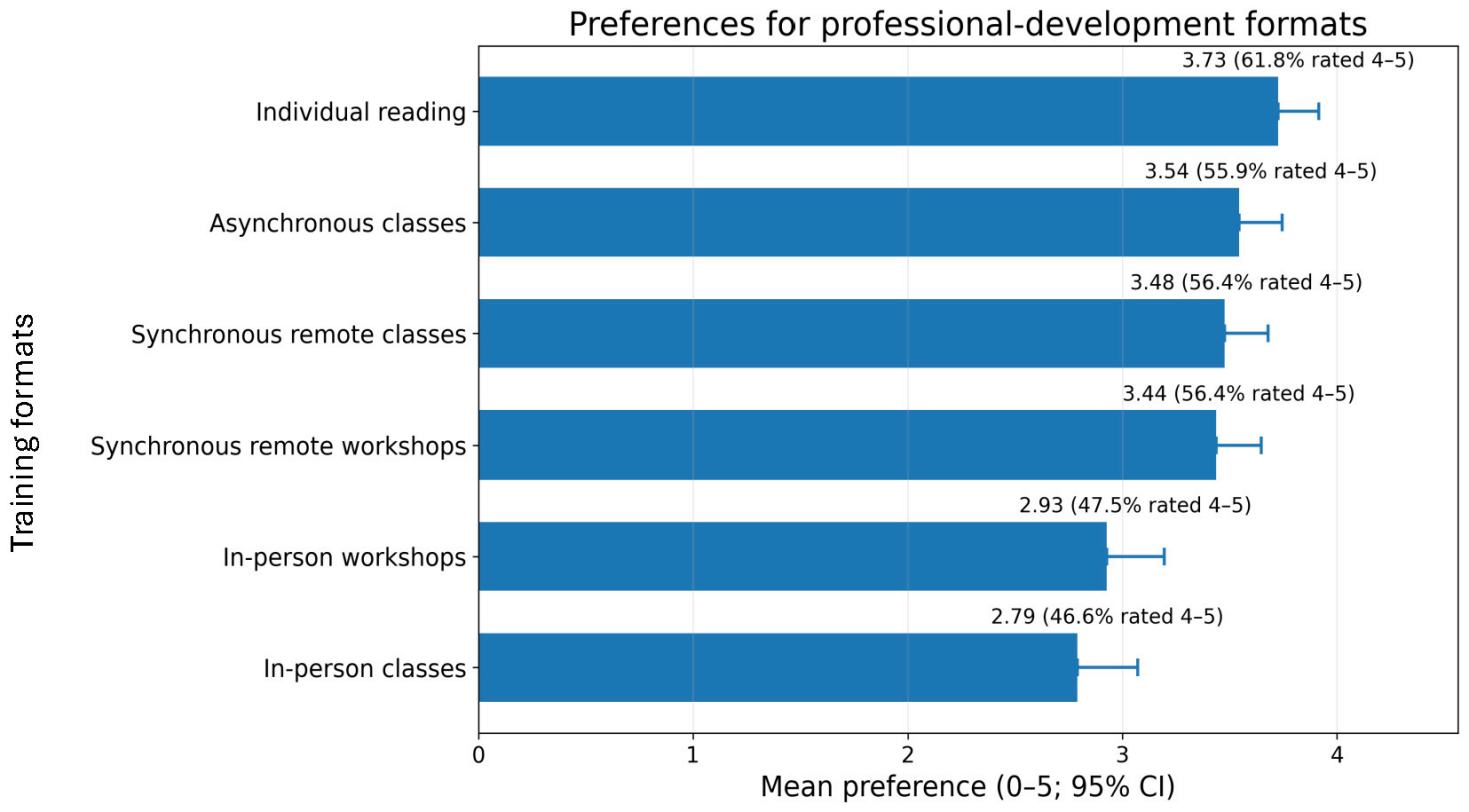
Preferences for professional-development formats. Error bars represent 95% confidence intervals; percentages indicate ratings of 4 or 5.

Preferences differed significantly across training formats [Friedman χ^2^ (5) = 20.89, *p* < 0.001], although the overall effect size was small (Kendall's *W* = 0.020). Individual reading received the highest mean rating (3.73), followed by asynchronous classes (3.54), synchronous remote classes (3.48), and synchronous remote workshops (3.44). In-person workshops (2.93) and in-person classes (2.79) received lower ratings. *Post-hoc* tests indicated that individual or remote formats were preferred to one or both in-person formats, whereas differences among remote formats were not significant. Training-format ratings and the proportion assigning scores of 4 or 5 are summarized in Figure 4.

## 4 Discussion

This secondary analysis should be understood as making three related, but distinct, contributions. First, it confirms the strong interest in neuroscience that has been repeatedly observed among teachers. Second, it extends prior work by examining, within each participant, the discrepancy between self-perceived current knowledge and desired future knowledge. Third, it translates that discrepancy into a practical ranking of content areas and delivery formats for professional development. The principal finding was therefore not simply that participants valued educational neuroscience, but that desired knowledge exceeded perceived current knowledge across all 10 topics, with a mean global discrepancy of approximately one point on a 0–5 scale and a positive global gap in nearly 70% of the sample.

The first level of contribution is consistent with a substantial literature showing that educators are interested in brain-based explanations of learning and development, even when their objective knowledge is incomplete and neuromyth endorsement remains common. Brazilian and international studies have documented the coexistence of enthusiasm for neuroscience with persistent misconceptions, including beliefs about learning styles, hemispheric dominance, and limited brain use (Dekker et al., 2012; Torrijos-Muelas et al., 2021; Simões et al., 2022; Adiguzel et al., 2025). The present results confirm this general pattern of high perceived relevance and motivation for further learning. Direct numerical comparison of the one-point gap with previous studies is not possible, however, because most earlier investigations assessed objective knowledge, neuromyth endorsement, or general interest rather than the same within-person difference between desired and self-perceived knowledge.

The second contribution is the use of a within-person discrepancy measure. Prior studies have generally treated knowledge, misconceptions, confidence, and interest as separate outcomes. By pairing current and desired ratings for the same participant and topic, the present analysis identifies areas in which motivation to learn exceeds perceived preparedness. This does not establish an objective competence deficit, and the gap should not be interpreted as equivalent to performance on a neuroscience test. Rather, it adds a complementary, learner-centered dimension to needs assessment by indicating where teachers themselves perceive the greatest distance between their current and desired states. In this sense, the findings extend, rather than directly replicate, the literature on neuroscience literacy and neuromyths.

The third contribution is practical prioritization. Neurodevelopment and brain plasticity produced the largest discrepancy. This result is coherent with the central place that development and plasticity occupy in attempts to connect neuroscience with education, but it also highlights an area in which scientifically accurate training is especially important. Concepts such as sensitive periods, experience-dependent change, and developmental variability are educationally relevant, yet simplified accounts of plasticity can encourage deterministic or commercially attractive claims that exceed the evidence. Brazilian discussions of teacher education have likewise emphasized the need to integrate neuroscience into professional preparation without reducing pedagogy to biological explanations (Grossi et al., 2024). Thus, the priority assigned to this topic supports training that addresses both the scientific foundations of plasticity and the limits of its classroom interpretation.

The adolescent brain, dyslexia/dysgraphia/dyscalculia, learning disorders, and ADHD also ranked highly. Their prominence is plausible because these topics correspond to recurrent educational demands involving attention, behavioral regulation, literacy and numeracy difficulties, developmental variability, and decisions about referral or support. Previous Brazilian research has shown that neuroscience knowledge and misconceptions vary across educational contexts and that teachers frequently encounter claims linking brain function to learning and behavior (Simões et al., 2022; Menezes, 2022). The present data extend that evidence by showing that these clinically and developmentally salient themes are not only areas of possible misunderstanding but also areas in which teachers report a comparatively large desire for additional knowledge. Nevertheless, the survey did not assess how often participants encountered each problem in practice, so the proposed connection with classroom challenges should be regarded as an informed interpretation rather than a directly tested explanation.

Memory and learning and socioemotional skills showed smaller discrepancies because self-perceived knowledge was comparatively higher, while desired knowledge remained high. This pattern may reflect greater exposure to these concepts in teacher education, professional-development activities, and public educational discourse, but the present dataset cannot determine the source of that familiarity. Accordingly, the result is better interpreted as showing a narrower perceived distance between current and desired knowledge, not as evidence that competence in these areas is sufficient. The continued high desired ratings suggest that both topics remain appropriate components of a core curriculum.

Reading scientific articles and critical scientific literacy produced the smallest mean gap. This should not be interpreted as indicating low importance. Scientific literacy is essential precisely because interest in neuroscience can coexist with difficulty distinguishing robust evidence from persuasive but unsupported claims (Dekker et al., 2012; Torrijos-Muelas et al., 2021). Brazilian work on teacher preparation has also emphasized engagement with scientific articles and other forms of evidence-based communication as part of professional literacy (Magalhães, 2023). A smaller perceived gap may reflect higher confidence, lower perceived immediacy, or underestimation of the complexity of critical appraisal. Therefore, scientific literacy should remain transversal to all topic modules rather than being deprioritized solely on the basis of the difference score.

The absence of meaningful differences in the global gap across school sectors, teaching levels, age, experience, gender, and disciplinary background also requires interpretation in relation to prior evidence. Brazilian studies have described substantial heterogeneity in working conditions, professional trajectories, and access to continuing education across public and private systems and across teaching contexts (Oliveira et al., 2016; Lemos and Fernandes, 2022; Langrafe Junior et al., 2025). Simões et al. (2022) additionally identified subgroup variation in neuroscience knowledge and neuromyth endorsement.

The present analysis does not contradict those findings, because it asks a different question: whether the overall within-person discrepancy between perceived and desired knowledge is concentrated in particular demographic or institutional groups. It was not. The near-zero effect sizes for school sector and teaching level and the low explanatory capacity of the multivariable model indicate that these measured characteristics contributed little to explaining the global perceived gap in this sample.

This lack of subgroup differentiation supports broad access to a common educational-neuroscience core, but it should not be taken to imply identical needs. Different teachers may reach the same global gap through different topic profiles, and the convenience sample may have preferentially attracted educators already interested in neuroscience or professional development. A modular strategy is therefore more defensible than either a single uniform course or programs restricted to selected demographic groups. A shared core could include scientific literacy, neurodevelopment, learning and memory, and explicit examination of neuromyths, with optional modules tailored to educational level, disciplinary role, and locally identified classroom demands.

The preference for individual reading and remote formats offers a further practical extension of the literature by connecting content priorities with delivery preferences. This pattern is compatible with the logistical realities of Brazilian teachers, including workload, variable institutional support, and unequal access to continuing education (Oliveira et al., 2016; Langrafe Junior et al., 2025). However, the extremely small Kendall's W shows that the statistically significant differences among formats were modest and should not be interpreted as strong or universal preferences. The findings support flexibility rather than replacement of in-person training. A blended program could combine concise asynchronous modules and guided readings with synchronous, case-based discussion and optional in-person activities.

The literature also suggests that delivery format alone is insufficient. Interventions addressing neuromyths are more effective when they explicitly refute misconceptions, explain why the incorrect claim is appealing, and require repeated application of accurate concepts to educational situations (Ferreira and Rodríguez, 2022; Lithander et al., 2024). Cross-national evidence further indicates that neuromyths remain widespread despite general familiarity with neuroscience terminology (Adiguzel et al., 2025). Accordingly, a gap-informed curriculum should integrate prioritized content with critical appraisal, explicit misconception correction, and realistic classroom cases rather than relying on factual lectures alone.

The training-gap indicator has practical advantages because it is transparent, directional, and easy to calculate at the individual, topic, or group level. It may help organize curriculum planning and can be repeated after an intervention to examine whether perceived need decreases, persists, or shifts toward more advanced content. At the same time, it is a descriptive difference score rather than a validated measure of objective competence. Identical differences may arise from different combinations of perceived and desired knowledge, and the score may be influenced by confidence, motivation, social desirability, or interpretation of the response scale. It should therefore be examined together with its two component ratings and, in future studies, with objective neuroscience-literacy and neuromyth measures.

Several limitations qualify the interpretation of these findings. The convenience recruitment through professional networks and social media limits generalizability and creates a risk of self-selection by teachers already interested in neuroscience. Regional information was not analyzed, all measures were self-reported, and no objective knowledge test was included in the same analytic framework. The cross-sectional design cannot establish whether the preferred formats would increase participation or improve learning. Moreover, because previous studies generally used different outcomes, the present gap values cannot be benchmarked directly against national or international norms. Finally, this is a secondary analysis of a previously collected survey: its distinct contribution lies not in a new sample, but in integrating paired ratings into a within-person discrepancy framework and using that framework to prioritize content and implementation options. Future research should preregister the metric, assess the reliability of its components and of the resulting score, compare it with alternative discrepancy models, recruit larger multi-regional samples, and test whether gap-informed curricula improve objective knowledge, critical appraisal, and classroom decision-making.

This secondary analysis introduces a simple training-gap metric that combines teachers' self-perceived current knowledge with their desire for additional learning. The principal finding was not merely that teachers were interested in educational neuroscience, but that desired knowledge systematically exceeded perceived current knowledge across every assessed topic. The mean discrepancy of approximately one point on a 0–5 scale, together with a positive global gap in nearly 70% of participants, indicates a broad perceived need for professional development.

The strongest priority was neurodevelopment and brain plasticity. This topic is foundational because it provides a conceptual framework for understanding developmental change, learning-related adaptation, sensitive periods, and the limitations of deterministic interpretations of brain development. Its high priority is especially relevant because simplistic claims about plasticity are frequently used to market educational interventions. Training should therefore address both the scientific basis of plasticity and the limits of its educational interpretation.

The adolescent brain, specific learning disorders, learning disorders more broadly, and ADHD also ranked highly. These themes are closely connected to classroom challenges that teachers encounter in everyday practice: behavioral regulation, attention, literacy and numeracy difficulties, developmental variability, and decisions about referral. High demand for these topics may reflect teachers' need for practical frameworks that help them recognize warning signs without converting educators into diagnosticians. Professional development should therefore clarify the educational role of teachers, the limits of classroom observation, and the importance of interdisciplinary assessment.

Memory and learning and socioemotional skills showed somewhat smaller gaps because baseline self- perceived knowledge was higher. Nevertheless, desired knowledge exceeded four points on average, indicating that these topics remain attractive. By contrast, reading scientific articles produced the smallest gap. This finding should not be interpreted as evidence that scientific literacy is unimportant. A modest gap can arise when current knowledge is perceived as relatively high, when the topic is seen as less immediately applicable, or when participants underestimate the complexity of critical appraisal. Given the persistence of neuromyths, scientific literacy should remain a transversal component of any curriculum (Dekker et al., 2012; Torrijos-Muelas et al., 2021; Simões et al., 2022).

Memory and learning and socioemotional skills showed smaller gaps in this sample because the arithmetic difference was reduced by comparatively higher self-perceived knowledge ratings. The study did not assess the source of that perceived familiarity. Greater exposure through teacher education, professional experience, continuing education, or public discussion is possible, but these explanations remain hypotheses and should be examined directly in future research.

An important result was the absence of meaningful differences in the global gap across school sectors, teaching levels, age, experience, gender, and disciplinary background. The related primary report identified subgroup differences in specific knowledge and interest items. The present analysis addresses a different question: whether the overall discrepancy between current and desired knowledge is concentrated in particular groups. It was not. The weak explanatory capacity of the multivariable model suggests that perceived demand for training is diffuse across the profession. Thus, broad access to core educational-neuroscience training may be more appropriate than restricting programs to selected demographic groups.

At the same time, a similar global gap does not imply that all teachers require identical content. Different subgroups may reach the same aggregate score through different topic profiles. A modular curriculum could therefore combine a common core—scientific literacy, neurodevelopment, learning and memory, and the critical evaluation of neuromyths—with optional modules tailored to educational level or professional role.

Format preferences provide guidance for implementation. Individual reading and remote formats were rated more highly than in-person formats. The small Kendall's W indicates that these preferences should not be treated as rigid categories, but the general pattern favors flexible delivery. Asynchronous resources may be particularly useful for teachers managing heavy workloads or limited institutional support, while synchronous remote sessions can preserve discussion and interaction. A blended program could pair short asynchronous modules and guided readings with live case-based seminars.

These findings also have implications for instructional design. Training should not be limited to factual lectures. Studies suggest that direct refutation, explanation of why a misconception is attractive, and repeated application to educational cases are more effective than passive exposure alone (Ferreira and Rodríguez, 2022; Lithander et al., 2024; Adiguzel et al., 2025). A needs-based curriculum should therefore connect prioritized topics to critical appraisal exercises, classroom scenarios, and explicit correction of common neuromyths.

The training-gap indicator has practical advantages. It is transparent, preserves the direction of discrepancy, and can be calculated at the individual, topic, or group level. It can also be repeated after an intervention to determine whether perceived need decreases, persists, or shifts toward more advanced topics. However, it remains a subjective measure. A high gap may represent low perceived knowledge, high motivation, or both, and it cannot substitute for objective knowledge testing.

Direct international benchmarks for this exact discrepancy score are not currently available, because most previous studies have examined objective neuroscience knowledge, neuromyth endorsement, or general interest rather than paired ratings of current and desired knowledge. The mean gap of approximately one point should therefore be interpreted internally: it corresponds to about 20% of the full 0–5 scale, was positive in nearly 70% of participants, and remained positive across all assessed domains. Future studies using the same paired-rating framework are needed to establish external reference values and cross-cultural comparability.

This study has limitations. The convenience sample limits generalizability, regional information was not analyzed, and all measures were self-reported. The survey did not include an objective neuroscience test in the same analytic framework, and perceived knowledge may be affected by overconfidence or under confidence. The cross-sectional design does not establish whether preferred formats lead to greater participation or learning. In addition, this report is a secondary analysis of the same survey dataset used in a related manuscript; its distinct contribution is the development and application of the training-gap framework rather than a new sample. Future studies should preregister the gap metric, recruit larger multi-regional samples, include objective literacy and neuromyth measures, and evaluate whether gap-informed curricula improve knowledge, critical appraisal, and classroom decision-making.

## 5 Conclusion

Brazilian teachers in this sample reported a consistent discrepancy between what they believed they knew about educational neuroscience and what they wished to learn. The largest perceived needs concerned neurodevelopment and brain plasticity, the adolescent brain, learning disorders, and ADHD. The global gap was broadly distributed and was not explained by school sector, teaching level, age, experience, gender, or disciplinary background. These results support a widely accessible professional- development strategy built around a common scientific core, prioritized topic modules, explicit correction of neuromyths, and flexible remote or reading-based formats. The training-gap indicator offers a practical tool for planning and evaluating such programs, provided that it is complemented by objective measures of knowledge and educational impact.

## Data Availability

The raw data supporting the conclusions of this article will be made available by the authors, without undue reservation.
